# Efficacy and Safety of 4.7 mg Deslorelin Acetate Implants in Suppressing Oestrus Cycle in Prepubertal Female Dogs

**DOI:** 10.3390/ani12243504

**Published:** 2022-12-12

**Authors:** Aymeric Gontier, Myriam Youala, Christelle Fontaine, Elsa Raibon, Sandrine Fournel, Philippe Briantais, Delphine Rigaut

**Affiliations:** 1 Research & Development, Licensing, Virbac, 06511 Carros, France; 2 Global Marketing & Market Development, Virbac, 06511 Carros, France

**Keywords:** castration, dog, female, heat prevention, neutering, oestrus suppression, postponement of puberty, prebuberty, reproduction, reversibility

## Abstract

**Simple Summary:**

In addition to convenience for owners, controlling female dog reproduction can be necessary to avoid unwanted pregnancy, especially at a young age. Surgical castration in a prepubertal bitch is still a controversial issue regarding the health benefits for the dog. Medical solutions, such as progestagens, often lead to severe side effects for the reproductive tracts. This is why temporary, reversible alternatives are required. Suprelorin^®^ (Virbac, Carros, France) is a slow-release implant based on deslorelin, a Gonadotropin-Releasing Hormone agonist. It was first labelled in male dogs for the induction of temporary infertility. This study evaluated the efficacy and safety of this contraceptive implant when administered in female dogs prior to puberty. The results confirmed that this method delays the onset of puberty for at least 5.5 months and, consequently, prevents unwanted and risky pregnancy in young female dogs.

**Abstract:**

Our multicentric, masked, controlled and randomised study aimed to assess the efficacy and safety of Suprelorin^®^ 4.7 mg (Virbac, Carros, France) regarding oestrus prevention in prepubertal intact bitches. Twelve- to eighteen-week-old females (n = 83) were allocated either a deslorelin implant (n = 62) or 0.9% sodium chloride (n = 21) group. Clinical assessment (heat signs), 17β oestradiol and progesterone assays, and vaginal cytology were performed at day (D)0, D7, D21, month (M)3 and M6 after product administration, and were then performed every other month until reaching puberty. Trained owners assessed heat signs between each veterinary visit. All bitches (n = 83) reached puberty before M30. Deslorelin significantly extended the median time to sexual maturity when compared to the control group (377 days versus 217 days after D0, *p* < 0.0001). Three females, implanted between 16 and 18 weeks of age, expressed an induced oestrus. Additional descriptive data, collected over a 24 month-period, showed functional reproductive abilities in both deslorelin (n = 52) and control (n = 21) groups once puberty was achieved. In conclusion, Suprelorin^®^ 4.7 mg seems to be an effective and safe option for postponing the onset of oestrus when administered to prepubertal female dogs aged from 12 to 16 weeks.

## 1. Introduction

Surgical castration rates in dogs tend to be highly variable, following cultural beliefs and habits. In the US, neutering privately owned males and females is considered a societal need to limit pet overpopulation issues [1]. Moreover, spaying bitches suppresses oestrus behaviour, vaginal discharge, and bleeding [1], along with the medical issues related to exposure to sexual hormones. In 2008, a statistical survey revealed that almost one third of the European female dog population was spayed (25% in France, 18% in Spain, 28% in Germany, and almost 67% in Switzerland), whereas less than 20% of the males were castrated (12%, 6%, 25%, and 32% in the same countries, respectively) [2]. On the contrary, another survey conducted in 2011 indicated that 78% of privately owned male and female dogs in the US had been neutered [1]. Those figures seemed to demonstrate that, despite cultural differences, some owners may remain reluctant to perform surgical neutering.

In females, prepubertal gonadectomy has frequently been advocated for instead of post-puberty spaying. Indeed, compared to post-puberty spaying, prepubertal gonadectomy induces a smaller wound, allows for a better organ visualisation, and requires a shorter recovery time after anesthesia [3]. Conversely, an increased incidence of post-operative bleedings, due to tissue disruptions, has been described after pre-pubertal gonadectomy performed before the age of 14 weeks in comparison to post-pubertal gonadectomy [4]. Nevertheless, it is commonly recognized that spaying bitches before the age of 2.5 years significantly reduces the risk of mammary carcinoma [5,6]. Spaying at an early age (especially younger than 3-months-old) may also lead to an under development of the vulva and to an increased risk of peri-vulvar dermatitis [7]. It is also important to check for the absence of juvenile vaginitis before spaying. This uncomplicated inflammation usually vanishes after the first heats, but may be persistent in case of prepubertal surgical castration [7]. The risk of urinary incontinence is still controversial, with some papers reporting an increased risk and more severe courses of incontinence when the spaying occurs before the first oestrus [7]. Epiphyseal fracture has not yet been proven to be an increased risk following spaying, although spaying significantly delayed epiphyseal closure in 7-week-old animals (compared to 7 months of age and to intact male and females) [7].

In the bitch, medical alternatives, such as progestagens, have been reported to induce modifications to the reproductive tract, such as pyometra, cystic endometrial hyperplasia, mammary tumours, polyphagia with weight gain, polydipsia due to sodium retention, diabetes mellitus, inhibition of endogenous cortisol secretion and hypersomatotropism related to an increased Insulin-like Growth Factor-1 mammary secretion [8]. Moreover, behaviour alterations, such as moderate lethargy or, conversely, restlessness, have also been reported [8]. Therefore, the benefit/risk balance of the use of progestagens remains controversial and their administration cannot be routinely advocated without a close monitoring of the animals.

Gonadotropin-Releasing Hormone (GnRH) agonists, such as deslorelin, are peptides whose structure presents similarities to GnRH, except at sites of enzymatic degradation. This allows for a better resistance to peptidases and enhances receptor-binding affinity. Therefore, GnRH agonists have a longer half-life in blood circulation [9]. The GnRH receptor found in mammals does not express a cytoplasmic C-terminal tail, as is usually observed with G-protein coupled receptors. The continuous exposure to a GnRH agonist instead leads to a post-receptor mechanism rather than to the internalisation of the GnRH receptor [10]. Contrary to the natural pulsatile secretion of GnRH, this sustained stimulation inhibits the synthesis of Luteinizing Hormone (LH) β- and Follicle Stimulating Hormone (FSH) β-subunits through a complex network of transduction pathways involved in gene expression. In the meantime, the α-subunit, which is common to both LH and FSH, is increased [9,10]. Following the LH and FSH inhibition, progesterone and 17β oestradiol (E2) are subsequently decreased [11].

Suprelorin^®^ 4.7 mg (deslorelin acetate, Virbac, Carros, France) has been commercialized for decades for the induction of temporary infertility in healthy, entire, sexually mature male dogs. In adult implanted female dogs, the temporary suppression of fertility may be preceded by the induction of oestrus. With a 4.7 mg deslorelin implant, the timeline between two oestrus has been described to vary from 2.1 to 27 months [11]. In intact prepubertal bitches, time to puberty was reported to range between 13 to 25 months after administration of a 4.7 mg deslorelin implant [12,13,14,15]. However, the published literature refers to studies run in experimental conditions [12,13,14] with treated groups of four [12,14] or eight animals [13]. This controlled study aimed to confirm, in field conditions, the efficacy and safety of the 4.7 mg deslorelin implant to delay puberty in female dogs; and to evaluate both the reversibility and return-to-normal of the reproductive function in deslorelin-implanted animals.

## 2. Materials and Method

### 2.1. Selection of Animals

This double-masked, controlled study was run in 3 French and 10 Spanish veterinary practices. A total of 83 healthy intact prepubertal bitches were enrolled in the study. Client-owned animals of any weight and breed were included, based on their age (from 12 to 18 weeks old), a normal genital appearance, and a normal ultrasound examination of the reproductive system (excluding any potential abnormalities that might have interfered with the objectives of the study) after the owner’s consent was signed. Animals presenting vaginitis, clinical or imaging signs of reproductive activity and animals having received sexual hormones since birth were not included. Furthermore, the use of any hormonal treatment during the trial that was intended for reproduction purposes led to animal exclusion. Ethical approval was given by the relevant ethical committee (EU-ERC/201703-03).

### 2.2. Study Products

On day (D)0, privately owned female dogs were randomly allocated to the 4.7 mg deslorelin acetate implant (Suprelorin^®^ Virbac, Carros, France, n = 62) group or to a negative-control group (1.0 mL of 0.9% sodium chloride; n = 21). Whatever the administered treatment, it was given subcutaneously between the shoulder blades, on D0, once the dog was included in the study.

### 2.3. Blinding

For masking purposes, the allocation, preparation and administration of study products were carried out by a dispenser who was different from the investigator at each veterinary practice (study site). The investigator was not present during the product administration. To ensure the blinding, laboratory personnel were not aware of the product received by the animal, and primary criteria were based on a quantitative result (i.e., blood hormonal dosages (E2 and progesterone (P4)) and vaginal cytology). The owners were also unaware of the treatment group of the animal.

### 2.4. Animal Follow-Up

The animals underwent complete physical examination, clinical assessment of heat signs, hormonal dosage (E2 and P4) and vaginal cytology at D0, D7, D21, month 3 (90 ± 2 days), and month 6 (180 ± 5 days) after inclusion. After month 6, similar assessments were then conducted every other month (60 ± 5 days) until the end of the study. Both the hormonal dosages and the vaginal smears were assessed by a central laboratory. The study completion was reached as soon as the onset of pro-oestrus or oestrus was observed and confirmed by laboratory results or, at the latest, by month 30 if the bitch was still prepubertal. The total blood count and biochemistry were obtained on D0 and at the last visit, when either pro-oestrus or oestrus was observed. Between each veterinary visit, owners were called and interviewed monthly to provide information on the absence, presence, or non-evaluation of the following heat signs: vulvar swelling, bloody vaginal discharge, restlessness, frequent urination, attraction of males, and mating acceptance. They were also instructed to signal any heat signs to the investigators as soon as they were detected. If a clinical sign was reported as present, an unscheduled visit was programmed as soon as possible to confirm the sexual maturity status through vaginal cytology and hormonal dosage. If the sexual maturity status was not confirmed by laboratory results, an additional visit was held approximately 15–20 days after the previous visit to run clinical and laboratory exams again. The owners were taught to identify these signs and return their animal to the clinic if they were detected. An additional sign, “immobility reflex”, was also required for veterinarians only. Some clinical signs that are not exclusively specific to heat manifestation, such as restlessness, which can indicate a playful or energetic puppy, or frequent urination also linked to urinary tract infections, were included in the evaluation grid to enhance any oestrus occurrence detection.

### 2.5. Evaluation Criteria

#### 2.5.1. Efficacy

The primary efficacy endpoint was defined as the time between treatment administration (D0) and sexual maturity, determined by laboratory results: blood hormonal dosages of E2, P4, and vaginal cytology [14]. Secondary efficacy endpoints included the mean E2 and P4 concentrations at the time of sexual maturity confirmation, the percentage of animals showing clinical signs of heat or silent heat (animals not showing any clinical signs of heat but confirmed to be sexually mature at reception of laboratory results), and the percentage of animals that never showed sexual maturity during the study.

The same methods and laboratories were used for all the samples. Serum P4 was measured using a chemiluminescence immunoassay which was processed on the Immulite 2000/Xpi system at Idexx laboratory, Ludwigsburg, Germany. The serum E2 concentrations were assessed by radioimmunoassay at Biocontrol laboratory, Imgelheim, Germany.

#### 2.5.2. Safety

The systemic safety and local safety were assessed by the investigator on the days when clinical examinations were performed until study completion. In addition, owners were instructed to observe their animals at least once daily and to immediately report any suspected adverse events to the investigator. If appropriate, the animal was brought back to the clinic for further examination.

#### 2.5.3. Long-Term Follow Up for Reversibility Assessment and Back-to-Normal Reproductive Function Evaluation

In order to evaluate both the reversibility of the implant effects and a return-to-normal reproductive function overtime, additional data on clinical reproductive parameters (heat cycles and pregnancies) were collected every 6 months after the first heat, and for a maximum period of 24 months. Various parameters were qualitatively evaluated regarding either the functional reproductive system (frequency of heats, number of puppies by litter, prolonged oestrous or galactorrhoea occurrence, and follicular resorption), or other parameters related to the effectiveness of the reproductive function (normality of the delivery, stillborn/alive puppies ratio, malformation, or abortion occurrences).

### 2.6. Statistical Analysis

The sample size was based on the application of a Log-rank test for comparison of the two “puberty curves”, with a median puberty time of 24 and 6 months in deslorelin and placebo groups, respectively (power at least 90%, alpha level: 5% two-sided), estimated based on the available literature [12,13,14]. The curve of time to onset of puberty was built using the Kaplan–Meier method and the distribution of time to onset was compared between treatment groups using a Log-rank test. The median time to onset was estimated for each group and reported. The comparison of the mean E2 and P4 concentrations at the time of sexual maturity between treatment groups was performed using a Wilcoxon’s rank sum test. The percentages of animals in each treatment group showing clinical signs of heat over time were compared using a Fisher’s exact test. For all statistical comparisons, significance was declared at a 5% two-sided level.

## 3. Results

### 3.1. Study Population

A total of 83 female dogs were enrolled. Seventy-seven animals were followed-up until the first oestrus apparition. Four female dogs were then lost to follow-up, which led to a total of seventy-three animals, which could be followed up after the first oestrus. Six animals were excluded from the study for reasons defined by the study protocol (Table S1): confirmation of sexual maturity within the 3 weeks following treatment (n = 3), serious or fatal adverse events unrelated to the treatment (n = 2, e.g., run over by a car and parvovirosis) and accidental ingestion of an unrelated sexual hormonal treatment (n = 1).

Efficacy data were calculated from eighty bitches, and three of them were considered until the time of their withdrawal (until the ingestion of the owner’s pill, death from parvovirosis infection, or death from a car accident). Safety data were obtained from the 83 enrolled bitches.

General characteristics at baseline were calculated from 59 and 21 bitches in the deslorelin and control groups, respectively, and showed similar conditions for both of them. The age at inclusion was 15.12 ± 1.79 weeks for the deslorelin group and 14.62 ± 1.86 weeks for the placebo group, ranging from 12 to 18 weeks in both groups. The mean bodyweight at inclusion was 8.2 ± 5.27 kg [min = 1 kg; max = 27 kg] in the deslorelin group and 8.5 ± 4.40 kg [min = 2 kg; max = 18 kg] in the control group. The ratio of mixed breed versus purebred was balanced between groups with a little over 50% of purebred in both groups. The breeds of the recruited female dogs and the proportion of these breeds within the deslorelin and the control group are presented in Table S2. The vaginal cytology (compatible with anoestrus) and hormonal dosages (basal E2 and P4 concentrations) confirmed that none of the dogs enrolled in the study had reached sexual maturity at baseline.

### 3.2. Time to Onset of Sexual Maturity

**Figure 1:**
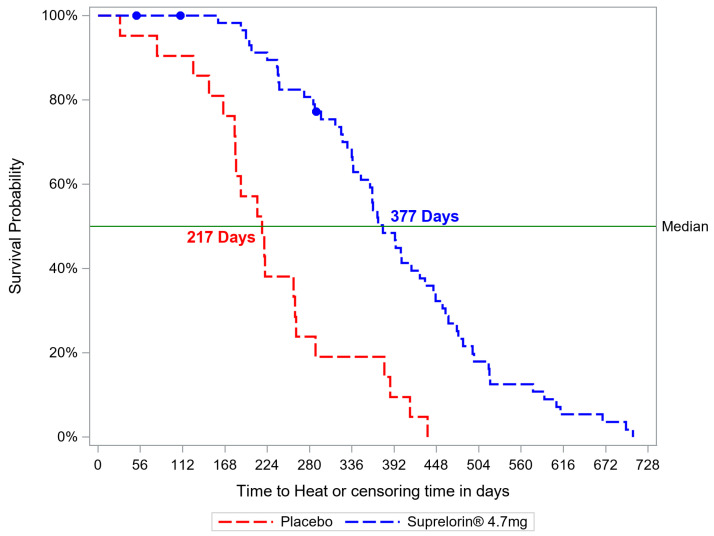
Time to heat occurrence in days over time, showing a significantly lower median in the placebo (red) than in the deslorelin (blue) group (*p* < 0.0001, 217 days vs. 377 days respectively).

The first oestrus onset ranged from 5.5 months to 24 months after implantation in the deslorelin group versus from 1 to 15 months in the control group. The curve of the time elapsed in days between D0 and sexual maturity occurrence for each animal (confirmed by a vaginal cytology different from anoestrus along with non-basal E2 and/or P4 blood concentration values) is represented in Figure 1, highlighting the individual variability. It has to be noted that data from any animal withdrawn from the study, due an adverse effect or forbidden medication occurrence, were taken into account up to the time of their withdrawal in this figure. The median time to sexual maturity after D0 was significantly prolonged in the deslorelin group compared to the control group (377 days versus 217 days respectively, *p* < 0.0001), which represented a delay of the onset of heat by over 5 months.

### 3.3. Evolution of E2 and P4 Blood Concentrations over Time

The hormonal levels of E2 and P4 were assessed on 56 and 21 dogs in the deslorelin group and control group, respectively.

One serum could not be analysed for E2 due to insufficient volume. Mean E2 concentrations at sexual maturity did not differ significantly at sexual maturity between groups (29.31 ± 15.69 pg/mL and 28.81 ± 11.65 pg/mL in the deslorelin and control groups, respectively, *p* = 0.9213).

In the same way, when reaching sexual maturity, mean P4 levels were comparable between treatment groups (3.47 ± 6.12 ng/mL and 2.12 ± 3.46 ng/mL in the deslorelin and control groups, respectively, *p* = 0.1874).

### 3.4. Percentage of Female Dogs with Signs of Heat at Different Time Points

Only signs of heat detected precisely at the scheduled time points could be used for statistical comparison between the two groups. At M6 and at M7, the percentage of dogs showing heat signs was significantly higher (*p* = 0.0094 and *p* = 0.0327) in the placebo group (18.8% and 16.7%, respectively) compared to the deslorelin group (0% and 0%, respectively). In the study protocol, reaching sexual maturity was a criterion to terminate the study. Thus, this led to a lowered number of remaining subjects in the untreated, placebo group over time. Therefore no statistical analysis could be performed after M8.

However, almost all the animals of both groups showed at least one clinical sign of heat at the time that sexual maturity was confirmed by laboratory results (98.2% and 100% in the deslorelin and placebo groups, respectively). Only one female dog in the deslorelin group was found to be sexually mature according to laboratory results, while no heat signs had been observed by the investigator during the last performed visit (M12). Further investigation showed that the owner actually missed some clinical and behavioural signs that were consistent with heat manifestation. Therefore, this case was reported as a deviation and not considered as a “silent heat”.

Finally, all the dogs that completed the study reached sexual maturity before the month 24 visit (Table S3).

### 3.5. Safety Results

Adverse events were reported for 31 bitches in the deslorelin group and 6 bitches in the control group, with no statistical differences in terms of incidence (Table S4). Overall, the most frequently reported adverse events (adverse events for which the incidence was over 5%) within the course of the study were, in the deslorelin group, lameness (12.9%), emesis (9.7%), dermatitis and eczema (8.1%), diarrhoea (8.1%) and vulvovaginitis (6.5%). In the placebo group, the most common adverse event was vulvovaginitis (19%). Four of the 8 cases of lameness had a traumatic etiology.

### 3.6. Long-Term Follow-Up for Reversibility of Implant Effect

Reproductive data were collected on the 73 animals (n = 52 in deslorelin group and n = 21 in placebo group) every 6 months and for a total length of a 24-month period observation, with the time limit set on October 2020.

Following the animals for an additional 24-month period allowed for up to 5 consecutive heat cycles to be observed in some of the bitches (Table 1). Some variability between individuals was noticeable in both groups, with a wide range of times observed in both groups (Table 1). The two groups were comparable regarding the frequency and regularity of the occurrence of heat signs (Table 1).

Intervals between clinical signs of heat and clinical patterns of oestrus cycles were similar between groups (Table 2). One animal from the deslorelin group was reported with an oestrus that lasted 24 days.

All the observed matings in the survey led to a pregnancy in both groups (Table 2). One female dog from the deslorelin group was inseminated and mated 9 months after its first heat. The first ultrasound performed at 5 weeks of pregnancy showed a live foetus and embryonic vescicles that had disappeared one week later. One live puppy was consequently born.

The use of the deslorelin implant did not affect the pregnancy rate after a return to fertility. All the puppies born from females treated with deslorelin were viable (Table 2), except one stillborn puppy belonging to a litter size of 10 (Great Dane breed). The litter sizes ranged between one to ten puppies in the deslorelin group. The gestating female dog of the control group gave birth to a litter of two puppies. The details of the reproductive performances are presented in Table S5. The percentage of animals presenting a galactorrhoea was similar in both groups (3.6% versus 4.8% in deslorelin and placebo groups, respectively, Table 2).

Finally, it has to be noted that no additional heat-, hormones- or pregnancy-related pathologies were observed in any female dogs during the 24 months of follow-up.

## 4. Discussion

In our study, the 4.7 mg deslorelin implants were proven to be an effective, reversible, and safe method to postpone the first signs of oestrus by 160 days in prepubertal female dogs. Regarding this onset of puberty, it has to be noted that the data found in the literature could not be easily compared. Indeed, both the populations of interest and the methodology often varied between studies. Using a 4.7 mg implant in 15 prepubertal bitches of different breeds, Maenhoudt et al. set their follow-up on the owner’s observations, with an oestrus confirmation assessed by a quantitative progesterone assay [16]. As a result, they observed eight dogs displaying the first oestrus at 13, 14, 15, 17, 20, 21, 23, and 24 months post-implantation, respectively. The six remaining bitches (implanted between 16 and 25 months before) did not show any sign of oestrus until the end of the observation period. In this study, Maenhoudt et al. measured the delay between the oestrus occurrence and the time of implantation. The lack of control group made the sexual maturity “delay” difficult to accurately estimate. Though sexual maturity could be affected by different factors (genetic factors, diet, housing conditions, etc.) small-breed females are expected to attain sexual maturity earlier than larger breeds [17]. These elements make the results difficult to be compared. Among medium-sized prepubertal subjects aged from 4 to 5.1 months at implantation, Kaya et al. reported a time to puberty at 72.7, 80.0, and >100.4 weeks of age for the 4.7 mg deslorelin-implanted female dogs (by mean, 84.4 weeks of age, n = 3), versus 53.0, 56.4, 63.4, and 75.0 weeks of age for the control bitches (by mean, 62.0 weeks of age, n = 4). Interestingly, such a delay (over 5 months) seemed to be consistent with our findings [12].

In our study, once sexual maturity was reached, clinical signs of heat did not differ between groups, confirming that the female dogs becoming sexually mature displayed normal heat signs after treatment with deslorelin. The clinical pattern of heats was also comparable to the control group: no silent heats or overexpression of clinical signs were observed in any of the groups. Sexual hormonal levels, once sexual maturity was reached, were also comparable in both groups, with no statistical differences between E2 and P4 means (*p* = 0.9213 and *p* = 0.1874, respectively). However, hormonal concentrations between individuals were highly variable, as shown by the large standard deviations. This variability could be explained by the field study constraints and, more specifically, the fact that animals were privately owned. Thus, it was not possible to standardise the sampling time corresponding to the first oestrus. Consequently, some female dogs were sampled in proestrus while others were sampled in oestrus. This can also explain why P4 levels (3.47 ± 6.12 ng/mL in deslorelin group and 2.12 ± 3.46 ng/mL in control group) were close to the theoretical lower limits expected during ovulation phase (ranging from 4.8 ± 0.9 ng/mL and 7.2 ± 1.3 ng/mL according to Hollinshead et al. [18]). However, despite this, the E2 and P4 mean concentrations were comparable between groups, indicating that the deslorelin implant did not change the hormonal pattern of the first oestrus. The frequency of the following heats was also comparable between groups, with a high individual variability inside the groups.

Implanting female dogs until 16 weeks of age seems to be a valuable option to avoid the “flare-up effect”. Indeed, in our study, when animals were implanted at 16 and 17 weeks of age, an oestrus was triggered before day 21 in three individuals in the deslorelin group. Previous studies had already reported an influence of the age at implantation in the response to the implant. In 7-month-old or older bitches, the 4.7 mg deslorelin implant triggered an oestrus in all dogs within 1–2 weeks. However, other works showed that 3- to 4-month-old pups presented no signs of oestrus during the 36 weeks of the experimental period [19,20]. In another controlled study, none of the 4-month-old, cross-bred female dogs implanted with a 4.7 mg deslorelin implant showed any clinical sign of oestrus [14]. Throughout the 13 weeks of observation period, only a slight increase of the superficial cell index (15–20%), accompanied by a moderate increase of E2 serum concentration (6–41 pg/mL) was reported in two dogs [14]. Similarly, Marino et al. were able to obtain oestrus suppression while implanting eight 4.5-month-old Sicilian hound females. In this experiment, where bitches were implanted three times every 4.5 months, only a modest increase of plasmatic E2 levels was measured 8 and 10 days after the first implant. A cornification of the vaginal mucosa cells was also noted [13]. Finally, it is assumed that the immaturity of the reproductive axis and the prepubertal genital tract would shut down the primary stimulating effects, thereby preventing the induced oestrus from occurring, contrary to what is commonly observed in the adult bitch [13].

Concurrently, the use of 4.7 mg deslorelin implants in 3- to 4-month-old prepubertal bitches was shown to be safe, whereas several side effects (metropathy, mucometra, persistent oestrus related or unrelated to ovarian cysts), probably due to the initial oetrogenic stimulation, were reported after the implantation of adult bitches [12,21]. Contrary to the findings of Marino et al. [13], where 4.5-month-old bitches, reimplanted every 4.5 months until 13.5 months of age, presented juvenile genitalia, such side effects were not observed in our study. Our conclusions are in line with the study from Kaya et al., in which medium-size, 4- to 5.1-month-old female dogs were randomly allocated to 9.4 mg, 4.7 mg deslorelin implants or to 0.9% sodium chloride; the size of the vulva, measured with a caliper, was not significantly different (*p* > 0.05) between the treatment and control groups for 40 weeks after inclusion [12]. Throughout the observation period, E2 levels remained actually basal, but not null, in implanted bitches. Kaya et al. hypothesized that this may allow for genitalia to develop [12]. On the contrary, early surgical spaying or close repeated implant insertions may suppress sexual hormone secretion more strongly, as suggested by the same research team. [12]. In this experiment, Kaya et al. also confirmed that implanted dogs showed a significantly delayed epiphyseal closure until 20 months of age compared to the placebo group; nevertheless, this had no clinical impact [12]. In the current study, lameness was described in eight implanted dogs. Four of these eight cases had a traumatic etiology. Moreover, seven cases were described as completely cured at the end of the observation period. The remaining uncompleted recovery was related to an accident, as the dog experienced a 3-meters-high fall. This tends to support the observation from Kaya et al. that delayed epiphyseal closure has no clinical significance. However, the evaluation of the impact of the implant on growth specifically, including the possible impact on the bodyweight at adulthood, was not the objective of this trial and would require specific investigations.

Once sexual maturity was reached, we observed a similar frequency in both treatment groups (3.6% in the deslorelin group and 4.8% in the placebo group) of animals presenting a galactorrhoea. Such a syndrome is due to the physiological drop in blood P4 levels by the end of the dioestrus phase, which is also associated, in some female dogs, with a release of prolactin. Galactorrhoea is, therefore, a consequence of the physiological hormonal pattern of the sexual maturity in dogs and should not be considered as a side effect [22].

In a recent study, many transcript levels of several factors were determined in the corpus luteum of control and implanted bitches, including: GnRH, oestrogen, progesterone, prolactin, and vascular endothelial growth factors receptors. The results enhanced the suggestion that this long-term delay of puberty did not seem to modify ovarian functionality in bitches [23].

From a long-term perspective, after a single administration of 4.7 mg implant, neither body weight, growth, nor fertility are expected to be affected by the puberty’s postponement, as previously described with other long-term-release GnRH agonist treatments, such as azagly-nafarelin [12]. Throughout the study and during the 24 months of follow-up after the appearance of the first oestrus, no reproductive-related side effects were reported in our study. Thus, the previously implanted female dogs had regular oestrus, with comparable clinical features to the negative control group, i.e., the females could breed and went on to conceive and give birth to viable litters. Our results are consistent with previous work, where Borges et al. estimated that fertility parameters such as ovulation or pregnancy rates in deslorelin-exposed animals did not differ from what was observed in untreated animals [24]. In our study, the litter sizes were consistent with the breed size of the female dog, except for one dog in each group. In the deslorelin group, a Wire-haired Daschund gave birth to a single puppy. In the control group, an English Setter delivered two puppies. Those figures are lower than expected regarding the weight of both female dogs [25]. This event was observed in both groups with the same occurrence.

These elements tended to confirm the short-term and the long-term safety of the 4.7 mg implant use in 3–4-month-old prepubertal bitches. Due to field conditions, female dogs were regularly lost for follow-up after return to fertility, which tended to decrease the number of evaluated dogs. Indeed, many of them were spayed, and thus, their reproductive capacities could not be observed. It could be interesting to pursue the evaluation of deslorelin on reproduction at a larger scale and from a longer-term perspective.

Among the reported adverse events, even though not statistically relevant, a localized alopecia at the site of the implant administration in one female dog was evaluated to be potentially related to the tested product. In the other cases, no link with deslorelin implant could be established. Although non-inflammatory alopecia or hair coat modification (“puppy coat”) [26] are reported with surgical castration or in specifications of the 4.7 mg deslorelin implant, the currently described cases of dermatitis and eczema do not seem to be related to the product use.

Gastro-intestinal signs, such as diarrhoea or emesis, found in the tested group were not previously described in the literature nor related to the mode of action of deslorelin. Therefore they could not be attributed to deslorelin.

Overall, more female dogs (n = 31) were reported to have side effects in the deslorelin group compared to control group (n = 6 females). This difference was not statistically relevant in term of incidence as more dogs were enrolled in the deslorelin group (n = 32) than in the control group (n = 21).

In our study, the implant was administered subcutaneously in the loose skin between shoulders, as in the experiment of Kaya et al. [12]. However, the implant was inserted in the post-umbilical region in other reports [11,16], to ease the removal when used for oestrus induction, to shorten the duration of action or in case of side effects. It is interesting to note that, in our study, the removal of the implant due to side effects did not occur, nor was it required. In other species, the implantation site was not reported to affect the efficacy or the safety of the product [27,28].

Vaginal smears were performed in the recruited females to confirm the prepubertal status and the absence of vaginitis. All the recruited females (aged from 13 to 18 weeks) presented a vaginal smear compatible with a prepubertal state. As a visual examination of the vulva should already offer some insight regarding vaginitis diagnosis, the vaginal smear, as a complementary examination, would, therefore, not be required in field conditions.

The protocol of our study was designed to assess the efficacy and safety of one single deslorelin implant administration in prepubertal female dogs. The follow-up observation period provided insights up to 24 months after the appearance of the first oestrus. Even though very encouraging, observations over a larger scale population and on a longer time period could be complementary and interesting. Additional data on consecutive implantations would also be very valuable in practice.

## 5. Conclusions

In this multicentric, randomized placebo-controlled study, 98.2% of prepubertal female dogs aged from 12 to 18 weeks and receiving a 4.7 mg deslorelin implant expressed their first oestrus from 6 to 24 months post implantation. The median onset of oestrus was significantly delayed by 160 days when compared to the control group. No safety concerns, including induced-oestrus, were observed in female dogs that were implanted until 16 weeks of age. At sexual maturity, implanted and control female dogs displayed comparable clinical heat signs and sex hormones levels. Reproductive data collected in the long-term (up to two years after the appearance of the first oestrus) support the hypothesis that the deslorelin-implanted female dogs returned to fertility, displayed regular heat cycles, were able to breed, and went on to conceive and to give birth without any major safety concerns. Therefore, it can be concluded that a single administration of a 4.7 mg deslorelin acetate implant (Suprelorin^®^ 4.7 mg, Virbac, France) seems to be an effective and safe option to postpone the onset of oestrus when administered to prepubertal female dogs aged from 12 to 16 weeks of age. This reversible neutering solution would offer owners more time to make an informed decision while preventing the risk of pregnancy at a young age.

## Figures and Tables

**Table 1:** Long-term (up to 24 months) qualitative observations of heat occurrence over time for the animals in the deslorelin and placebo groups, after the occurrence of the first oestrus. * Oestrus signs displayed 2–4 weeks outside of the 24-month observation period. SD = standard deviation. N/A = not applicable. n = number of animals (% within group).

Heat Occurrence over Time	Time since the Previous Heat (Range and Mean Values in Months)		
	Parameters	Deslorelin Group	Placebo Group
Second heat	Range [min–max] Mean (±SD)	[3.9–11.3]	[2.6–10.6]
		6.7 (±1.8)	6.7 (±2.5)
		n = 26 (50.0%)	n = 11 (52.4%)
Third heat	Range [min–max] Mean (±SD)	[3.3–12.7]	[4.1–10.1]
		7.6 (±2.8)	5.9 (±2.0)
		n = 11 (21.2%)	n = 7 (33.3%)
Fourth heat	Range [min–max] Mean (±SD)	[5.4–7.1]	[5.5–8.6]
		6.2 (±0.8)	6.6 (±1.4)
		n = 4 (7.7%)	n = 4 (19.1%)
Fifth heat	Range [min–max] Mean (±SD)	N/A	[6.5–7.0]
		7.2 (N/A)	6.8 (±0.4)
		n = 1 (1.9%)	n = 2 * (9.5%)

**Table 2:** Description of the reproductive performances in the animals of the deslorelin and placebo groups over up to 24 months after the occurrence of the first heat.

	Deslorelin Group	Placebo Group
Successful mating or artificial insemination	9/9	1/1
Total number of pregnancies (1st heat–2nd heat)	9 (3–6)	1
Litter size range	1–10	2
Stillborn/alive puppies	1/34	0/2
Malformation	0	0
Abortion	0	0
Prolonged oestrus	1	0
Galactorrhoea	2	1
Follicular resorption	1	0

## Data Availability

The data are not publicly available. Specific requests for additional information can be sent to the corresponding author at christelle.speiser-fontaine@virbac.com.

## Supplementary Materials

The following are available online at https://www.mdpi.com/article/10.3390/ani12243504/s1, Table S1. Parameters of the inclusion and non-inclusion criteria for the animals entering the study protocol, along with the withdrawal criteria. Table S2. Proportion of the breeds within each group (deslorelin and control) reported at inclusion. Table S3. Duration of effect of the deslorelin implant when assessed as the primary efficacy endpoint: the effect was reversible, with a maximum duration of 708 days in the deslorelin group (23 months maximum) vs. 436 days in the placebo group (14 months max.). Table S4. Adverse events reported in the deslorelin and control groups. Table S5. Details of the reproductive performances over up to 24 months after the occurrence of the first heat.
