# Supplementary material for: Efficacy and Safety of 4.7 mg Deslorelin Acetate Implants in Suppressing Oestrus Cycle in Prepubertal Female Dogs
Source: Animals (Basel). 2022 Dec 12;12(24):3504. doi: 10.3390/ani12243504 (PMC9774346; doi:10.3390/ani12243504)
Supplement: Supplementary file 1 [file animals-12-03504-s001.zip › animals-1932913-supplementary.pdf]

animals

MDPI

Articles

Efficacy and safety of 4.7mg deslorelin acetate implants in suppressing oestrus cycle in prepubertal female dogs.

Aymeric Gontier[1], Myriam Youala[1], Christelle Fontaine[2], Elsa Raibon[2,*], Sandrine Fournel[1], Philippe Briantais[1] and Delphine Rigaut[1]

Supplementary material

Table S1. Parameters of inclusion and non-inclusion criteria for the animals entering the study protocol and withdrawal criteria.

Criteria	Characteristics used in the study protocol
**Inclusion criteria**	Obtention of owner's informed consent (prior to the start of the study)<br>Pre-pubertal intact healthy female dogs<br>Dogs aged between 12 and 18 weeks<br>Normal genital appearance (e.g. no vulvar discharge)<br>Normal ultrasound examination of the reproductive system<br>Animals maintained in a controlled environment that allows for daily observation of pro-oestrus or oestrus (i.e. heat) sign<br>Availability of the animals for the entire duration of the study
**Non-Inclusion criteria**	Vulvar deformity<br>Vaginitis<br>Reproduction-related hormonal administration (since birth)<br>Sexually mature animal prior to treatment administration confirmed by clinical or ultrasound examination (e.g. pro-oestrus or oestrus signs, pregnancy)<br>Any concomitant disease that may interfere with the evaluation of the treatment<br>Any animal owned by an Investigator or Dispenser involved in the study
**Withdrawal criteria (during study)**	Occurrence of an adverse event which may interfere with the evaluation of the study treatment<br>Detection of a sexually mature animal (i.e. silent heat or clinical heat) anytime between D1 and Day 21 post-treatment administration<br>Confirmation of sexual maturity prior to treatment administration upon reception of Day 0 laboratory results<br>Abnormal laboratory results in the pre-treatment sample<br>Sterilization becoming mandatory for the animal during the study<br>Major protocol deviation (from the owner or the investigational team)<br>Withdrawal of the Owner's consent

Table S2. Proportion of the breeds within each group (deslorelin and control) reported at inclusion.

Reported Breed at Inclusion	Deslorelin group (n=59)	Placebo group (n=21)
American bully- exotic	-	4.8% (n=1)
American Pit Bull Terrier	3.4% (n=2)	-
American Staffordshire terrier	1.7% (n=1)	-
Andalusian rat - hunting dog	1.7% (n=1)	-
Australian Shepherd	1.7% (n=1)	-
Beagle	1.7% (n=1)	-
Beauceron	1.7% (n=1)	-
Boston terrier	1.7% (n=1)	-
Boxer	3.4% (n=2)	-
Chihuahua	1.7% (n=1)	-
Corso Dog	-	4.8% (n=1)
Czechoslovakian Wolfhound	1.7% (n=1)	-
English Setter	5.1% (n=3)	14.3% (n=3)
Fila Brasileiro	-	4.8% (n=1)
French Bulldog	1.7% (n=1)	-
German Shepherd	1.7% (n=1)	14.3% (n=3)
German Shorthaired Pointer	3.4% (n=2)	-
Golden Retriever	1.7% (n=1)	-
Great Dane	1.7% (n=1)	-
Labrador Retriever	3.4% (n=2)	-
Malinois	1.7% (n=1)	-
Maltese	-	4.8% (n=1)
Miniature Pinscher	-	4.8% (n=1)
Miniature Schnauzer	1.7% (n=1)	-
Parson Russell Terrier	3.4% (n=2)	4.8% (n=1)
Petit Basset Griffon Vendeen	3.4% (n=2)	4.8% (n=1)
Podengo	1.7% (n=1)	9.5% (n=2)
Poodle*	5.1% (n=3)	-
Pug	1.7% (n=1)	-
Samoyed	1.7% (n=1)	-
Shih Tzu	1.7% (n=1)	-
Siberian Husky	1.7% (n=1)	-
Spanish Greyhound	1.7% (n=1)	-
Spitz Papillon	1.7% (n=1)	-

Wirehaired Dachshund	1.7% (n=1)	-
Wirehaired Pointing Griffon	-	4.8% (n=1)
Yorkshire Terrier	3.4% (n=2)	-
% of purebred vs. mixed bred	54.2% vs. 45.8%	52.4% vs. 47.6%
Not specified breed	28.8% (n=17)	23.8% (n=5)

* can be either a Poodle, a Miniature Poodle or a Toy Poodle.

Table S3: Duration of effect of the deslorelin implant when assessed as the primary efficacy endpoint: the effect was reversible over time, with a maximum duration of 708 days in the deslorelin group (23 months maximum) vs. 436 days in the placebo group (14 months maximum).

**Time interval for sexual maturity occurence**		**Sexually matured animals deslorelin group (n=59)**	**Placebo group (n=21)**
[0 - 6] months post implantation	N of animals<br>% within the group	n=4 *<br>6.7%	n=6<br>28.6%
[6 - 9] months post implantation	N of animals<br>% within the group	n=9<br>15.3%	n=10<br>47.6%
[9 - 12] months post implantation	N of animals<br>% within the group	n=16<br>27.1%	n=1<br>4.8%
[12 -18] months post implantation	N of animals<br>% within the group<br>Maximum duration	n=23<br>39.0%	n=4<br>19.0%<br>436 days
[18 - 24] months post implantation	N of animals<br>% within the group<br>Maximum.duration	n=7<br>11.9%<br>708 days	n=0<br>0.0%
> 24 months post implantation	N of animals<br>% within the group	n=0<br>0.0%	n=0<br>0.0%

* details for these animals: three dogs, implanted over 16 weeks of age, expressed an induced oestrus after implantation. For one dog, the duration of effect was 5.5 months.

Table S4: Adverse events reported in the deslorelin and control groups.

	**Deslorelin group (n=62)**		**Placebo group (n=21)**		**p-value**
	**Number of animals**	**% within group**	**Number of animals**	**% within group**	
**Lameness**	8	12.9	-	-	-
**Emesis**	6	9.7	1	4.8	-
**Dermatitis and eczema**	5	8.1	-	-	-
**Diarrhoea**	5	8.1	-	-	-
**Vulvovaginitis**	4	6.5	4	19.0	-
**Abnormal oestrus***	3	4.8	-	-	-
**Lethargy**	3	4.8	-	-	-
**Unclassifiable adverse event**	3	4.8	-	-	-
**Anorexia**	2	3.2	-	-	-
**Cough**	2	3.2	-	-	-
**Dehydration**	2	3.2	-	-	-

	Deslorelin group (n=62)		Placebo group (n=21)		p-value
	Number of animals	% within group	Number of animals	% within group	
**Gastritis**	2	3.2	-	-	-
**Joint pain***	2	3.2	-	-	-
**Pruritus**	2	3.2	-	-	-
**Trauma***	2	3.2	1	4.8	-
**Aggression**	1	1.6	-	-	-
**Alopecia**	1	1.6	-	-	-
**Bacterial skin infection**	1	1.6	-	-	-
**Bronchitis**	1	1.6	-	-	-
**Cartilage degeneration**	1	1.6	-	-	-
**Conjunctivitis**	1	1.6	1	4.8	-
**Death**	1	1.6	-	-	-
**Dermal cyst(s)**	1	1.6	-	-	-
**Dermal mass**	1	1.6	-	-	-
**Dyspnoea**	1	1.6	-	-	-
**Fracture**	1	1.6	-	-	-
**Haemorrhagic diarrhoea**	1	1.6	-	-	-
**Hyperactivity**	1	1.6	-	-	-
**Hyperthermia**	1	1.6	-	-	-
**Infarction**	1	1.6	-	-	-
**Localised pain***	1	1.6	-	-	-
**Myopathy**	1	1.6	-	-	-
**Nail disorder***	1	1.6	-	-	-
**Neuromuscular disorder***	1	1.6	-	-	-
**Oedema***	1	1.6	-	-	-
**Otitis externa**	1	1.6	-	-	-
**Otitis***	1	1.6	-	-	-
**Pica***	1	1.6	-	-	-
**Polydipsia**	1	1.6	-	-	-
**Polyuria**	1	1.6	-	-	-
**Sexual disorder***	1	1.6	-	-	-
**Skin oedema**	1	1.6	-	-	-
**Tachycardia**	1	1.6	-	-	-
**Tachypnoea**	1	1.6	-	-	-
**Anxiety**	-	-	1	4.8	-
**Cataract**	-	-	1	4.8	-
**Corneal ulcer**	-	-	1	4.8	-
**Genital tract pruritus**	-	-	1	4.8	-
**Lens disorder***	-	-	1	4.8	-
**Sneezing**	-	-	1	4.8	-
**Uveitis**	-	-	1	4.8	-
**Total of animals with at least one adverse event**	31	50.0	6	28.6	0.0877

* No further details provided by the investigators or owners

Table S5: Details of reproductive performances over up to 24 months after the occurrence of the first heat.

Group	Breed	Time period after the first heat	Litter size (number of puppies)	Observations
Placebo	English setter	6 months	2	
Deslorelin	Great dane	First heat	9 + 1 stillborn	
Deslorelin	Parson russell terrier x basset griffon vendeen	First heat	5	
		13.5 months	N/A	The female died in labor.
Deslorelin	Pitbull terrier	First heat	7	
		7 months	N/A	The female was spayed at one month of gestation to end this unwanted pregnancy.
Deslorelin	Beagle	8 months	6	
Deslorelin	Wire-haired dachshund	9 months	1	The female was inseminated and mated.
Deslorelin	Pitbull terrier	7 months	N/A	The female was spayed at one month of gestation to end this unwanted pregnancy.
Deslorelin	French bulldog	2 years	6	Caesarean.

Tableau mis en forme
